# Plasminogen activator inhibitor type 1 regulates microglial motility and phagocytic activity

**DOI:** 10.1186/1742-2094-9-149

**Published:** 2012-06-29

**Authors:** Hyejin Jeon, Jong-Heon Kim, Jae-Hong Kim, Won-Ha Lee, Myung-Shik Lee, Kyoungho Suk

**Affiliations:** 1 Department of Pharmacology, Brain Science & Engineering Institute, CMRI, Kyungpook National University School of Medicine, 101 Dong-In, Daegu, Joong-gu, 700-422, South Korea; 2 School of Life Sciences and Biotechnology, Kyungpook National University, Daegu, South Korea; 3 Department of Medicine, Samsung Medical Center, Sungkyunkwan University School of Medicine, Seoul, South Korea

## Abstract

**Background:**

Plasminogen activator inhibitor type 1 (PAI-1) is the primary inhibitor of urokinase type plasminogen activators (uPA) and tissue type plasminogen activators (tPA), which mediate fibrinolysis. PAI-1 is also involved in the innate immunity by regulating cell migration and phagocytosis. However, little is known about the role of PAI-1 in the central nervous system.

**Methods:**

In this study, we identified PAI-1 in the culture medium of mouse mixed glial cells by liquid chromatography and tandem mass spectrometry. Secretion of PAI-1 from glial cultures was detected by ELISA and western blotting analysis. Cell migration was evaluated by *in vitro* scratch-wound healing assay or Boyden chamber assay and an *in vivo* stab wound injury model. Phagocytic activity was measured by uptake of zymosan particles.

**Results:**

The levels of PAI-1 mRNA and protein expression were increased by lipopolysaccharide and interferon-γ stimulation in both microglia and astrocytes. PAI-1 promoted the migration of microglial cells in culture via the low-density lipoprotein receptor-related protein (LRP) 1/Janus kinase (JAK)/signal transducer and activator of transcription (STAT)1 axis. PAI-1 also increased microglial migration *in vivo* when injected into mouse brain. PAI-1-mediated microglial migration was independent of protease inhibition, because an R346A mutant of PAI-1 with impaired PA inhibitory activity also promoted microglial migration. Moreover, PAI-1 was able to modulate microglial phagocytic activity. PAI-1 inhibited microglial engulfment of zymosan particles in a vitronectin- and Toll-like receptor 2/6-dependent manner.

**Conclusion:**

Our results indicate that glia-derived PAI-1 may regulate microglial migration and phagocytosis in an autocrine or paracrine manner. This may have important implications in the regulation of brain microglial activities in health and disease.

## Introduction

Activated glial cells secrete a variety of proteins including proinflammatory cytokines, chemokines, and neurotoxic factors under inflammatory or pathological conditions [1,2]. Secretomic analysis has been previously conducted for astrocytes [3-5] and microglia [6,7] to determine the profile of the secreted proteins. Some of these secreted proteins play important roles in the progression of inflammatory diseases in the brain, and serve as biomarkers that can be used to guide diagnosis and drug therapy. Microglia, the resident macrophages of the CNS, constitute the brain’s innate immune system and play a pivotal role in neuroinflammation and host defense against microbial agents [8-12]. Microglia, as phagocytes, engulf invaded pathogens, apoptotic cells, and their debris [11,13]. Chronically activated microglia also contribute to neurotoxicity in neurodegenerative diseases, such as Alzheimer’s disease (AD), Parkinson’s disease (PD), amyotrophic lateral sclerosis, Huntington’s disease, and multiple sclerosis (MS) [14-19]. Migration of microglia, via extension of their processes, to the site of inflammation is a key step in the progression of the inflammatory brain diseases [20].

Plasminogen activator inhibitor type 1 (PAI-1), also known as serine protease inhibitor E1, is expressed in various cell types such as adipocytes, glomerular mesangial cells, epithelial cells, vascular endothelial cells, vascular smooth-muscle cells, monocytes/macrophages, and astrocytes [21-23]. PAI-1 acts as the main inhibitor of both urokinase type plasminogen activators (uPA) and tissue type plasminogen activators (tPA), which convert plasminogen to plasmin. This plasmin activator/inhibitor system is involved in the regulation of fibrinolysis, and remodeling of the extracellular matrix, cell migration, and invasion of tumor cells [21,24-26]. PAI-1 is also involved in the distinction between viable and apoptotic cells, and PAI-1 regulates the phagocytosis of apoptotic cells [27]. PAI-1 plays a dual role in the regulation of cell migration through differential interactions with its binding partners such as uPA, tPA, vitronectin, and low-density lipoprotein receptor-related protein (LRP)1. The PAI–vitronectin complex binds to the Arg-Gly-Asp motif of αv integrins and inhibits the integrin-mediated cell migration [28-33]. The PAI-1/uPA/uPAR complex inhibits uPA-induced cell migration [34], whereas the interaction between PAI-1 and LRP1 stimulates the movement of monocytes [35-37]. The LRP1/tPA/PAI-1 complex induces Mac-1-dependent macrophage migration [37]. Thus, the effect of PAI-1 on cell migration depends on the binding proteins involved, which are expressed in a cell- and tissue-specific manner. Overexpression of PAI-1 has been detected in various brain disorders, such as glioma, ischemic stroke, MS, and AD [18,38-42]. Several reports have indicated an important role of PAI-1 in the CNS injury and pathology. Increased PAI-1 was shown to interfere with the clearance and degradation of amyloid-β by blocking tPA, and inactivation of PAI-1 retarded the progression of AD pathology [39]. PAI-1 reduced brain edema and axonal degeneration after ischemic brain injury [42]. PAI-1 produced by astrocytes protected neurons against N-methyl-D-aspartate receptor-mediated excitotoxicity [43], and PAI-1 expressed in olfactory ensheathing glia was shown to promote axonal regeneration [44]. However, the role of PAI-1 in the regulation of microglial functions has not been investigated.

In the present study, we identified PAI-1 as a protein secreted from mixed glial cultures after stimulation with lipopolysaccharide (LPS) and interferon (IFN)-γ. PAI-1 levels were increased in both microglia and astrocytes by inflammatory stimulation. Subsequent studies showed that glia-derived PAI-1 specifically regulated microglial cell motility. Using LRP1 small interfering (si)RNA and low-density lipoprotein receptor-associated protein (RAP), we found that PAI-1 promoted microglial migration through an LRP1-dependent mechanism. Further examination of the signaling pathways indicated that the PAI-1/LRP1 complex enhanced microglial migration via the JAK/STAT1 pathway. The migration-promoting effect of PAI-1 did not require the PA inhibitory activity, either *in vitro* or *in vivo*. In addition, we found that PAI-1 inhibits microglial phagocytic activity. Studies using PAI-1 mutant proteins indicated that the inhibitory effect of PAI-1 on microglial phagocytosis was dependent on vitronectin but not LRP1. Taken together, our results suggest that PAI-1 may be released predominantly by microglia and astrocytes under inflammatory conditions of the brain, and the secreted PAI-1 protein may regulate microglial migration and phagocytosis in CNS inflammation.

## Methods

The animals used in this study were maintained under temperature- and humidity-controlled conditions with a 12 hour light/12 hour dark cycle. All animal experiments were approved by the institutional review board of Kyungpook National University School of Medicine and were carried out in accordance with the guidelines in the NIH *Guide for the Care and Use of Laboratory Animals*.

### Reagents

LPS (from *Escherichia coli* 0111: B4 prepared by phenolic extraction and gel filtration chromatography), BSA, and rabbit serum were all purchased from Sigma (Sigma, St Louis, MN, USA). Recombinant mouse IFN-γ, RAP protein, and recombinant human vitronectin protein were purchased from R&D Systems (Minneapolis, MN, USA). Lipoteichoic acid (LTA) from *Bacillus subtilis* was purchased from InvivoGen (Carlsbad, CA, USA). 5-chloromethyl-fluoresceindiacetate (CMFDA) was purchased from Molecular Probes Inc (Eugene, OR, USA). JAK inhibitor AG490 ((E)-*N*-benzyl-2-cyano-3-(3,4-dihydroxyphenyl) acrylamide a-cyano-(3,4-dihydroxy)-N-benzylcinnamide tyrphostin B42), was purchased from Calbiochem (La Jolla, CA, USA). Recombinant mouse PAI-1 protein was purchased from American Diagnostica (Greenwich, CT, USA), and was diluted in PBS. All other chemicals, unless otherwise stated, were obtained from Sigma.

### Preparation of recombinant human PAI-1 proteins

The bacterially expressed recombinant human PAI-1 wild-type and mutant proteins (Q123K and R346A) were prepared as previously described [45]. The PAI-1 mutant Q123K was unable to bind to vitronectin [16,46,47], and the R346A mutant was unable to inhibit PA [28,45]. In brief, the coding region of recombinant wild-type human PAI-1 (amino acids 24–402, Swiss-Prot primary accession number P22777) was cloned into the pRSET B vector with an N-terminal polyhistidine (6 × His) tag (kindly provided by Dr Hana Im, Sejong University, Seoul, Korea) [47]. This PAI-1 construct lacks the N-terminal secretory signal region. Human PAI-1 mutants were generated by using a site-directed mutagenesis kit (QuikChange; Stratagene, La Jolla, CA, USA) in accordance with the manufacturer’s instructions. The pRSET B vector containing the wild-type or mutant PAI-1 cDNA was transformed into the competent *E. coli* strain BL21(DE3) pLysS, which was then grown at 37°C in 500 ml of Luria broth medium supplemented with 100 μg/ml ampicillin. The expression of recombinant proteins was induced with 0.1 mmol/l Isopropyl β-D-1-thiogalactopyranoside (IPTG) for 3 hours, and then cells were lysed by sonication. The protein was purified by using nickel-nitrilotriacetic acid (Ni-NTA) beads (Qiagen, Chatsworth, CA, USA) in accordance with the manufacturer’s instructions. Ni-NTA-bound proteins were then eluted with an buffer containing 50 mmol/l Tris–HCl (pH 8.0), 100 mmol/l NaCl, and 200 mmol/l imidazole. The purified protein was dialyzed (Slide-A-Lyzer Dialysis Cassettes; Pierce, Rockford, IL, USA), and then concentrated using centrifugal dialysis filtration tubes (Millipore, Billerica, MA, USA).

### Cell cultures

The BV-2 mouse microglial cell line, which exhibits phenotypic and functional properties comparable with those of primary microglial cells [48,49], was grown and maintained in DMEM containing 5% FBS, 2 mmol/l glutamine, penicillin, and streptomycin (Gibco, Gaithersburg, MD, USA) at 37°C in 95% air/5% CO_2_. C6 rat glioma cells were grown and maintained under the same condition as the BV-2 microglial cells. Primary mixed glial cells and astrocyte cultures were prepared as previously described [50]. In brief, the forebrains of newborn ICR mice were chopped and dissociated by mechanical disruption using a nylon mesh.

The cells were seeded into culture flasks. Mixed glial cultures were established after *in vitro* culture for 10 to 14 days at 37°C in 95% air/5% CO_2_. Mixed glial cultures were composed of 61.86 ± 1.44% astrocytes, 28.73 ± 2.23% microglia, and 9.36 ± 1.92% ‘other cell types’ as determined by glial fibrillary acidic protein (GFAP) and ionized calcium binding adaptor molecule 1 (Iba-1) staining [51]. Astrocytes were isolated from mixed glial cultures by shaking at 270 rpm for 2 hours. This resulted in the detachment of microglia, whereas astrocytes remained attached to the bottom of the culture flask. The detached microglia were aspirated, and the remaining astrocytes were used for experiments. Astrocyte cultures were composed of 92.56 ± 3.14% astrocytes, 0.45 ± 1.0% microglia, and 6.99 ± 2.23% other cell types as determined by GFAP and Iba-1 staining.

Primary microglial cultures were separately prepared by mild trypsinization as previously described [52] with minor modifications. After *in vitro* culture for 10 to 14 days, microglial cells were isolated from mixed glial cultures by mild trypsinization. Mixed glial cultures were incubated with a trypsin solution (0.25% trypsin, 1 mmol/l EDTA in Hank’s balanced salt solution) diluted 1:4 in PBS containing 1 mmol/l CaCl_2_ for 30 to 60 minutes. This resulted in the detachment of the upper layer of astrocytes in one piece, whereas microglia remained attached to the bottom of the culture flask. The detached layer of astrocytes was aspirated, and the remaining microglia were used for experiments. The purity of microglial cultures was greater than 95% as determined by GFAP and Iba-1 staining. Primary glial cultures were grown and maintained in DMEM supplemented with 10% FBS, 100 U/ml of penicillin, and 100 μg/ml of streptomycin.

Primary cultures of dissociated cerebral cortical neurons were prepared as previously described [53]. Cortical neurons were grown in neurobasal medium containing 10% FBS, 0.5 mmol/l glutamine, 100 U/ml of penicillin, 100 μg/ml of streptomycin, N2 supplement (Gibco), and B27 supplement (Gibco). The purity of the neuronal cultures was determined by immunocytochemical staining, using an antibody against a neuron-specific marker, microtubule-associated protein 2 (Promega, Madison, WI, USA).

### Proteomic analysis of conditioned medium of mixed glial cultures

Conditioned medium from mixed glial cultures or cell extracts was prepared as previously described [51]. Cells were treated with a combination of LPS (100 ng/ml) and IFN-γ (50 U/ml) at 37°C in 95% air/5% CO_2_ for 24 hours. The stimulation was performed under serum-free conditions. Precipitated proteins were analyzed by liquid chromatography and tandem mass spectrometry (LC-MS/MS) as previously described [51,54].

### Traditional reverse transcriptase PCR and real-time PCR

Total RNA was extracted from cells by using TRIzol reagent (Invitrogen), in accordance with the manufacturer’s protocol. Reverse transcription was conducted using reverse transcriptase (Superscript II; Invitrogen) and oligo(dT) primers. PCR amplification, using specific primer sets, was carried out at an annealing temperature of 55 to 60°C for 25–30 cycles. The PCR was performed in a thermal cycler (DNA Engine Tetrad Peltier; MJ Research, Waltham, MA, USA). For the analysis of PCR products, 10 μl of each PCR was separated by electrophoresis in 1% agarose gels and viewed under UV light. β-actin was used as an internal control. Nucleotide sequences of the primers were based on published cDNA sequences of mouse LRP-1, PAI-1, Toll-like receptor (TLR)2, TLR6, dectin-1, and β-actin (Table 1)

**Table 1:** List of PCR primers used in the study

**Name**	**Accession number**		**Forward primer (5**′**→3**′**)**
**Reverse transcriptase PCR**			
LRP1	NM_008512	Forward	GCGTGGGTGTGTGATGGCGA
		Reverse	GTTGCGCAGGCCAGGCACTA
PAI-1	NM_008871	Forward	GTCTTTCCGACCAAGAGCAG
		Reverse	GCCGAACCACAAAGAGAAAG
TLR2	NM_011905.3	Forward	ACAGCTACCTGTGTGACTCTCCGCC
		Reverse	GGTCTTGGTGTTCATTATCTTGCGC
TLR6	NM_011604.3	Forward	CTGCCCTGGTATGTGAGGAT
		Reverse	TCTGGATGAAGTGGGGAGAC
Dectin-1	AY534909.1	Forward	GACTTCAGCACTCAAGACATCC
		Reverse	TTGTGTCGCCAAAATGCTAGG
β-actin	NM_007393.3	Forward	ATCCGTAAAGACCTCTATGC
		Reverse	AACGCAGCTCAGTAACAGTC
**Real-time PCR**			
PAI-1	NM_008871	Forward	CATGCCCCACTTCTTCAAGCT
		Reverse	TGGTATGCCTTTCCACCCAGT
GAPDH	NM_008084.2	Forward	TGGGCTACACTGAGCACCAG
		Reverse	GGGTGTCGCTGTTGAAGTCA

LRP, Low-density lipoprotein receptor-related protein; PAI, plasminogen activator inhibitor; TLR, Toll-like receptor.

Real-time PCR was performed using a commercial kit (One Step SYBR®PrimeScript^TM^; Takara Bio Inc, Japan) in accordance with the manufacturer’s instructions, followed by detection using the ABI Prism® 7000 Sequence Detection System (Applied Biosystems, Foster City, CA). Nucleotide sequences of the primers were based on published cDNA sequences of mouse PAI-1 and mouse GAPDH (Table 1), the latter used as an internal control.

### Western blotting analysis

Western blotting analysis was carried out as previously described [51]. In brief, cells were treated with LPS (100 ng/ml), IFN-γ (50 U/ml), or mouse PAI-1 (100 ng/ml) at 37°C in 95% air/5% CO_2_. Cells were washed with PBS and lysed in triple-detergent lysis buffer (50 mmol/l Tris–HCl (pH 8.0), 150 mmol/l NaCl, 0.02% sodium azide, and 1% NP-40). After SDS-PAGE separation of the cell lysates, proteins were transferred to nitrocellulose membranes (Amersham Biosciences, Piscataway, NJ, USA). The membranes were blocked with 5% skim milk, and sequentially incubated with primary antibodies (rabbit polyclonal anti-mouse PAI-1 antibody, anti-LRP1 antibody (H-80; raised against the N-terminal extracellular domain of 515 kDa LRP1) (both Santa Cruz Biotechnology, Santa Cruz, CA, USA), rabbit polyclonal anti-STAT1 antibody, rabbit polyclonal anti-phospho-STAT1 antibody (both Cell Signaling Technology, Beverly, MA, USA), monoclonal anti-mouse TLR2 antibody, monoclonal anti-mouse TLR6 antibody monoclonal anti-mouse TLR9 antibody (all Imgenex, San Diego, CA, USA), or monoclonal anti-α-tubulin clone B-5-1-2 mouse ascites fluid (Sigma)), and horseradish peroxidase (HRP)-conjugated secondary antibodies (anti-mouse IgG (Amersham Biosciences) and anti-rabbit IgG (Cell Signaling Technology)), followed by ECL detection (Amersham Biosciences).

### Indirect ELISA for plasminogen activator inhibitor type 1

Indirect ELISA was used for the recombinant mouse PAI-1 protein measurements. Cells were treated with a combination of LPS (100 ng/ml) and IFN-γ (50 U/ml) for 24 hours. The stimulation was performed under serum-free conditions. The conditioned medium was then collected, and separated by centrifugation at 400 g for 5 minutes to remove cell debris. The wells of microtiter plates were coated with conditioned medium overnight (diluted 1:1 in 50 mmol/l carbonate buffer, pH 9.6; 100 μl/well in triplicate wells). Plates were washed with PBS plus 0.1% Triton X-100 and blocked with PBS plus 5% BSA for 1 hour. Plates were emptied, and any remaining liquid was tapped out onto dry paper towels. Rabbit polyclonal anti-mouse PAI-1 antibody was added (1 μg/ml; 100 μl per well) and incubated for 5 hours. Plates were washed three times with PBS-T to remove unbound antibody. Horseradish peroxidase-labeled anti-rabbit IgG was added (1:1000 dilution; 100 μl per well) and incubated for 1 hour. Plates were washed three times with PBS-T and developed by the addition of 100 μl of tetramethylbenzidene peroxide-based substrate solution (R&D Systems). The recombinant mouse PAI-1 protein was used as a standard.

### Nitrite quantification

Cells were seeded at the density of 5 × 10^4^ cells/well in 96-well plates, and treated with various stimuli for 24 hours in serum-free medium. Production of NO was estimated by measuring the amount of nitrite, a stable metabolite of NO, using Griess reagent, as previously described [51].

### Assessment of cell viability and proliferation

Cells (5 × 10^4^ cells in 100 μl/well for cell viability assay; 5 × 10^3^ cells in 100 μl/well for cell proliferation assay) were seeded in 96-well plates and treated with various stimuli for the specific time periods in the serum-free medium. After treatment, 3-[4,5-dimethylthiazol-2-yl]-2,5-diphenyltetrazolium bromide (MTT) assay was performed as previously described [51].

### Microglia/neuron co-culture

For the co-culture of microglia and neurons, primary microglia were seeded at a density of 4 × 10^4^ cells/well in 96-well plates at 37°C in 95% air/5% CO_2_. After 16 hours of incubation, the cells were treated with LPS (100 ng/ml) and mouse PAI-1 protein (100 ng/ml) for 12 hours. Culture medium was then removed, and cells were washed with PBS. CMFDA-labeled mouse primary cortical neurons (4 × 10^4^ cells/well) were added to microglia-plated wells and incubated in neurobasal medium containing 10% FBS. After an additional 24 hours incubation period, the number of CMFDA-labeled cells was counted at ×100 magnification in four visual fields in each well using a fluorescence microscope (CK2; Olympus, Tokyo, Japan). Images of five random fields per well were captured and analyzed by an imaging system (MetaMorph; Universal Imaging Corp, West Chester, PA) [53].

### Cell migration assays *in vitro*

Cell migration was determined by using an *in vitro* scratch-wound healing assay or Boyden chamber assay. The scratch-wound healing assay was performed as previously described [55,56]. In brief, BV-2 mouse microglial cells and C6 rat glioma cells were seeded at a density of 8 × 10^4^ cells/well in 96-well plates, and incubated at 37°C under in 95% air/ CO_2_ for 14 hours. A scratch wound was created with a 200 μl pipette tip on the confluent cell monolayer. Cells were treated with or without pharmacological inhibitors, PAI-1 protein, BSA, RAP protein, astrocyte-conditioned medium (ACM), anti-PAI-1 antibody, or rabbit serum. Cells were allowed to recover for 24 hours in serum-free medium. The wound closure was then viewed under a microscope (Olympus CK2; original magnification, × 150). Relative cell migration distance was determined by measuring the wound width and subtracting this from the initial value:

fold increase of migration distance = (sample initial wound width at time 0 minus sample wound width at 24 hours of measurement)/(control initial wound width at time 0 minus control wound width at 24 hours of measurement).

A total of three areas were selected and examined in each well. The fold increase of migration distance was based on the average wound width in three areas (an area with maximum width, an area with minimum width, and a randomly chosen area). The results were presented as the fold increase of the migration distance compared with control.

A 48-well Boyden chamber (NeuroProbe, Gaithersburg, MD, USA) was also used for the measurement of cell migration, in accordance with the manufacturer’s instructions. The recombinant mouse PAI-1 protein in DMEM containing 10% FBS was placed into the lower wells, which were separated from the upper wells by polyvinylpyrrolidone-free polycarbonate filters (8 μm pore size, 25 × 80 mm; NeuroProbe). Primary microglial cells were harvested by trypsinization, resuspended in serum-free DMEM, and added to the upper chamber at a density of 5 × 10^4^ cells/well. Cells were incubated at 37°C under in 95% air/5% CO_2_ for 24 hours. At the end of the incubation, any non-migrating cells on the upper side of the membrane were removed with a cotton swab. Migrated cells on the lower part of the membrane were fixed in methanol for 10 minutes and stained with Mayer’s hematoxylin (Dako Cytomation, Glostrup, Denmark) for 20 minutes. Photomicrographs of five randomly chosen fields were taken (Olympus CK2) (original magnification, × 100), and cells were enumerated to calculate the average number of cells that had migrated. All migrated cells were counted (MetaMorph imaging system; Molecular Devices). Results are presented as the mean ± SD of triplicates.

### Small interfering RNA transfection

Control siRNA and mouse LRP1 siRNA pool (CGCUGACCCUAUUUGAAGAtt, UCUUCAAAUAGGGUCAGCGtt; CCUUCAGCAUCGAUGUGUUtt,AACACAUCGAUGCUGAAGGtt; CUACCUACAAGAUGUAUGAtt, UCAUACAUCUUGUAGGUAGtt)were purchased from Santa Cruz Biotechnology. siRNA transfection of BV-2 microglial cells was performed (Lipofectamine^TM^ 2000; Invitrogen) in accordance with the manufacturer’s instructions. The cells were harvested 48 hours after transfection, and used for the experiments.

### Dot blotting analysis

Cells were treated with LPS (100 ng/ml), IFN-γ (50 U/ml), or mouse PAI-1 protein (100 ng/ml). Cells were then washed with PBS and lysed in triple-detergent lysis buffer (50 mmol/l Tris–HCl (pH 8.0), 150 mmol/l NaCl, 0.02% sodium azide, 1% NP-40). Cell lysates were spotted slowly onto nitrocellulose membranes (Hybond ECL; Amersham Biosciences). The membranes were then blocked with 5% skim milk and sequentially incubated with anti-LRP1 antibody and HRP-conjugated anti-rabbit IgG followed by ECL detection.

### Astrocyte-conditioned medium

To prepare ACM, primary astrocyte cultures were seeded at the density of 1.5 × 10^6^ cells in 100 mm culture dishes. Primary astrocyte cultures were treated with a combination of LPS (100 ng/ml) and IFN-γ (50 U/ml) for 12 hours. Cells were then washed twice with PBS, and cultured in fresh DMEM for an additional 24 hours. The ACM was then collected, separated by centrifugation at 400 g for 5 minutes to remove cell debris, and stored at −80°C until further analysis.

### Intrastriatal injection of human plasminogen activator inhibitor type 1 (PAI-1) protein

Mice (body weight 30 g) were anesthetized by intraperitoneal injection of tiletamine/zolazepam (30 mg/kg (Zoletil; Virbac Laboratories, Carros, France)) and xylazine (10 mg/kg (Ropum; Bayer, Puteaux, France)), and positioned in a stereotaxic apparatus (Stoelting, Wood Dale, IL, USA). The mice were placed on a homeothermic heat blanket (Harvard Apparatus Co., South Natick, MA, USA) at 37°C to maintain normal body temperature during surgery. The skull was exposed by a skin incision, and a small hole was drilled through the skull. To avoid passing through the ventricles, the guide cannula was implanted at the stereotaxic coordinates of 1 mm anterior to the bregma, 2 mm lateral to the bregma, and 4 mm below the skull using a 22 G needle, and cemented. Intrastriatal injection of the vehicle (dialyzed elution buffer) or recombinant human PAI-1 protein of wild-type or R346A mutant (1 μl; 1.5 μg/μl) was performed using a 26 G needle. Denatured PAI-I protein, which was used as a control, was prepared by heating for 15 minutes at 95°C. The flow rate of the injection was 0.1 μl/min maintained by a microsyringe pump (Harvard Apparatus Co.). After removing the needle, the skin was sutured with 6.0 mm silk thread. The mice were killed 48 hours after the injection.

### Immunohistochemistry

Mice were anesthetized with ether, and transcardially perfused with 4% paraformaldehyde in PBS. Brains were post-fixed and cryoprotected with 30% sucrose solution for 24 hours. The fixed brains were embedded in optimal cutting temperature compound (Tissue-Tek; Sakura Fine-Tek, Tokyo Japan) and then cut into 12 μm-thick coronal sections on a cryostat. The tissues were permeabilized in 0.1% Triton X-100, and blocked with 1% BSA and 5% normal serum. After washing with PBS, the sections were incubated at 4°C overnight with rabbit polyclonal Iba-1 antibody (1:500 dilution; Wako, Tokyo, Japan). The sections were then incubated with biotinylated anti-rabbit IgG antibody (1:200 dilution; Vector Laboratories, Burlingame, CA, USA). Subsequently, the sections were incubated with avidin–biotin complex reagents (Vector Laboratories) for 30 minutes at room temperature, followed by detection with diaminobenzidine.

### Stab-injury and cell-injection assay

To evaluate *in vivo* microglial cell migration, we used a stab-wound injury model as described previously [57,58]. ICR mice (30 g) were anesthetized by intraperitoneal injection of tiletamine/zolazepam 30 mg/kg and xylazine 10 mg/kg, and positioned in a stereotaxic apparatus, on a homeothermic heat blanket at 37°C to maintain normal body temperature during surgery. The skull was exposed by a sagittal skin incision, and a small hole was drilled through the skull. The guide cannula was implanted at 4 mm lateral from the bregma, and 3 mm below the skull using a 22 G needle, and cemented. After 3 days, the skull bone located at 2 mm posterior from the guide cannula was thinned with a high-speed drill, and then a 3 × 2.5 × 0.1 mm sterilized razor blade was stereotaxically inserted to a depth of 3 mm below the skull to create a coronal stab injury, and immediately removed. After removing the blade, the bone was covered (Bone Wax; Ethicon Inc, Somerville, NJ, USA). Primary microglial cells (7.0 × 10^5^ cells) were incubated with 1 μg/ml of recombinant human PAI-1 proteins (denatured PAI-1 wild-type as a control, wild-type PAI-1, and PAI-1 mutant R346A) in six-well plates for 12 hours, and labeled with 5 μmol/l CMFDA for 15 minutes. Intracortical cell injection (together with 1 μl PAI-1 proteins at 1 μg/μl) was performed using a 26 G needle through a guide cannula with a flow rate of 0.1 μl/min using a microsyringe pump. After surgery, skin was sutured with 6.0 mm silk thread. At 72 hours after the injection, the mice were killed.

Migration of CMFDA-labeled microglial cells was estimated using immunofluorescence assay. Iba-1 immunofluorescence staining was performed as described above for immunohistochemistry, except the secondary antibody was donkey Cy3-conjugated anti-rabbit antibody (1:1000 dilution; Jackson Immunoresearch Laboratories, West Grove, PA, USA). The sections were mounted on gelatin-coated slides and allowed to air-dry overnight.

### Data acquisition and immunohistological intensity measurement

The level of coronal sections that passed the striatum was determined in accordance with a mouse brain atlas. Tiled images of each section were captured with a charge-coupled device color video camera (D70; Olympus) through a 100 × objective lens attached to a microscope (BX51; Olympus). A composite of the images (640 × 480 pixels each; 1 pixel = 2.5 × 2.5 μm) was then constructed for each section with Photoshop CS3. Immunohistological intensity analysis of Iba-1 staining was performed (Image J; NIH, Bethesda, MD, USA) as previously described [59]. Composite images of stained sections were fast Fourier transform band-pass filtered to eliminate low-frequency drifts (>20 pixels = 50 μm) and high-frequency noises (<1 pixel = 2.5 μm). The image was set with a binary threshold of 50% of the background level, and then the particles were converted to a subthreshold image area with a size of 20 to 300 pixels, which was judged as showing the Iba-1-positive cells. This range (20 to 300 pixels) was obtained from the analyzed size of Iba-1-positive cells from six sections for each animal. To count the Iba-1 positive cells, five squares (300 × 300 μm) were placed around the injection site in the subthreshold image of the six independent sections, and the cells in the five squares were counted and statistically analyzed.

### Phagocytosis of fluorescent zymosan particles

BV-2 microglial cells were seeded at a density of 7.5 × 10^4^ cells/well in 24-well plates. Cells were treated with the recombinant human PAI-1 protein (100 ng/ml), mouse PAI-1 protein (0.1 to 1 μg/ml), BSA (2.2 or 22.0 nmol/l), monoclonal anti-mouse TLR2 antibody, polyclonal anti-mouse integrin β3 (ITGB3) antibody (Cell Signaling Technology), and vitronectin (1 μg/ml) for 1 hour in serum-free DMEM. Cells were then incubated at 37°C for 3 hours with 30 μg/ml of fluorescent zymosan particles (zymosan A (*Saccharomyces cerevisiae*) BioParticles conjugated with Alexa Fluor 594; Molecular Probes Inc). Primary microglia cultures were similarly treated with mouse PAI-1 (100 ng/ml) or RAP (5 μg/ml) for 1 hour, and then incubated with 30 μg/ml of zymosan particles for 90 minutes. Cells were then washed five times with ice-cold PBS to remove bound particles. Photomicrographs of five randomly chosen fields were taken (CK2; Olympus) in three separate experiments. A minimum of 400 microglial cells per well were counted, and the percentage of phagocytic cells was determined as previously described [51]. Recent reports have indicated that washing three to five times with ice-cold PBS effectively removed extracellular bacteria [60] and zymosan particles [61,62]. We also determined by confocal microscopy whether bound particles could be removed by washing five times with ice-cold PBS. After phagocytosis assay, microglial cells with different focal planes were examined under a confocal microscope to visualize the uptake of fluorescent particles. The results indicated that repeated washes could remove surface-bound particles efficiently (data not shown).

### Statistical analysis

All data are presented as mean ± SD from three or more independent experiments, unless stated otherwise. Statistical comparisons between different treatments were performed by a Student’s *t*-test or one-way ANOVA with Dunnett’s multiple comparisons by using SPSS software (version 18.0; SPSS Inc, Chicago, IL). *P* < 0.05 was considered significant.

## Results

### Increase in plasminogen activator inhibitor type 1 (PAI-1) level in both microglia and astrocytes by inflammatory stimuli

Secreted proteins can regulate various cellular processes, such as cell growth, proliferation, cell death/survival, and homeostasis [63,64]. A large-scale analysis of glia-derived proteins may broaden the understanding of glial functions in the CNS [3,6,51]. We and others have previously investigated the secretome of brain glial cells [3,4,6,7,51,65,66]. Proteins secreted from glial cells have been shown to regulate neuron–glia communication and to play important roles in interglial interactions [67]. In the present study, we identified PAI-1 as the major secreted protein of glia through LC-MS/MS analysis of mouse mixed glial cultures. Primary mixed glial cultures were prepared from neonatal mouse brain and treated with LPS and IFN-γ for 24 hours. Conditioned medium was then subjected to LC-MS/MS analysis. PAI-1 secretion was strongly induced by LPS/IFN-γ treatment in the mixed glial cultures, with the number of peptide hits in unstimulated and LPS/IFN-γ-stimulated glia being 0 and 16, respectively.

**Figure 1:**
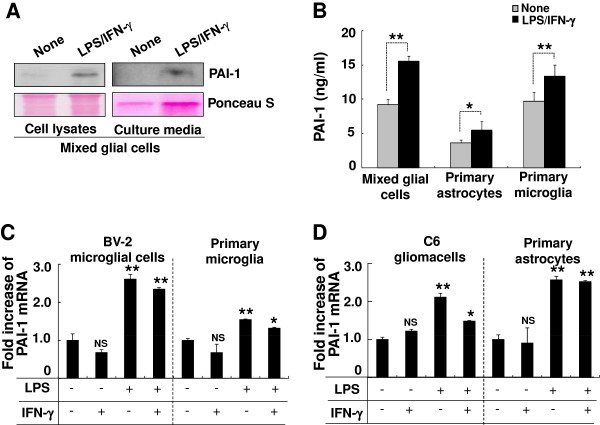
**Expression of plasminogen activator inhibitor type 1 (PAI-1) mRNA and protein in mixed glial cultures. (A)** Cell lysates or conditioned medium were prepared from either untreated or lipopolysaccharide (LPS)/interferon (IFN)-γ-treated mixed glial cultures (24 hours treatment). Western blotting analysis was conducted to evaluate the expression level of the 47 kDa PAI-1 protein. Ponceau S staining images are shown for comparison. **(B)** The mixed glial cells, microglia, and astrocytes were stimulated with a combination of LPS (100 ng/ml) and IFN-γ (50 U/ml) for 24 hours, and conditioned medium were collected. PA1-1 secretion was measured by ELISA. Results are given as mean ± SD (*n* = 3). *P < 0.05, ** *P* < 0.01. **(C, D)** Real-time PCR was performed to detect PAI-1 mRNA expression in glial cells treated for 6 hours with LPS (100 ng/ml) and IFN-γ (50 U/ml) either alone or in combination as indicated. GAPDH was used as an internal control. Results are given as mean ± SD (*n* = 3). *P < 0.05, ** *P* < 0.01, NS = not significant; compared with the untreated control cells.

PAI-1 secretion from mixed glial cells was verified by western blotting analysis using a specific antibody. The PAI-1 protein band of 47 kDa was detected in cell lysates and conditioned medium. LPS/IFN-γ increased PAI-1 protein expression was 4.63-fold in the glial lysates and 6.23-fold in the conditioned medium, respectively, when normalized to Ponceau S staining (Figure 1A). PAI-1 was barely detectable in the conditioned medium of unstimulated glial cell cultures, consistent with the LC-MS/MS data. Soluble proteins from conditioned medium were precipitated using TCA/acetone solution, and the precipitate was solubilized in a detergent-containing buffer. This method was used to detect the proteins of low abundance in LC-MS/MS and western blotting analyses. However, discrepancies in the protein precipitation and solubility may produce different protein profiles. For the direct quantification of PAI-1 levels in the conditioned medium and the identification of cellular source of PAI-1 secretion, PAI-1-specific ELISA was performed for the separate glial cell cultures. LPS/IFN-γ stimulation similarly increased the secretion of PAI-1 in the mixed glial cells, microglia, and astrocytes (Figure 1B), indicating that both microglia and astrocytes contribute to glial PAI-1 secretion. PAI-1 mRNA levels were also augmented by inflammatory stimulation in microglia and astrocytes. LPS, alone or in combination with IFN-γ, enhanced PAI-1 mRNA expression to varying degrees in glial cell lines and cultures (Figure 1C,D), but IFN-γ alone did not have a significant effect. These results indicate that both microglia and astrocytes can be the major cellular sources of PAI-1 in the CNS under inflammatory conditions.

### Plasminogen activator inhibitor type 1 (PAI-1) promotes microglial migration, but not microglial proliferation or neurotoxic activation

**Figure 2:**
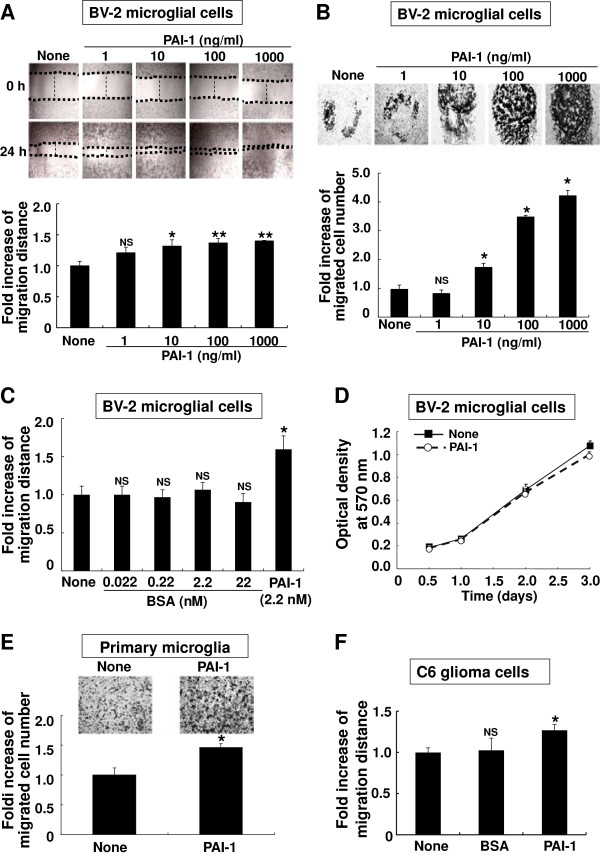
**Plasminogen activator inhibitor type 1 (PAI-1) promoted the migration of microglia in a concentration-dependent manner. (A)** BV-2 microglial cells were seeded at a density of 8.0 × 10^4^ cells/well in 96-well plates. When the BV-2 microglial cells had reached 80 to 90% confluence, a single scratch wound was made by using a 200 μl pipette tip, and the cell debris was removed by washing with PBS. Cells were treated with mouse PAI-1 protein (0 to 1000 ng/ml). At 0 and 24 hours, phase-contrast pictures of the wounds at three different locations were taken, and then the fold increase of migration distance was measured in three independent experiments. Results are given as mean ± SD (*n* = 3). *P < 0.05, NS = not significant, compared with the untreated control (lower panel). Representative images are shown (upper panel; original magnification,× 150). **(B)** The Boyden chamber assay was also performed to evaluate cell migration. BV-2 microglial cells (5 × 10^4^ cells/upper well) placed in the Boyden chambers were exposed to mouse PAI-1 protein (0 to 1000 ng/ml), and then incubated at 37°C for 6 hours. Microglial cells that migrated through a membrane were stained and counted. Results are given as mean ± SD (*n* = 3). *P < 0.01; compared with the untreated control (lower panel). Representative images are also shown (upper panel; original magnification, × 100). **(C)** BV-2 microglial cells were treated with BSA (0 to 22 nmol/l) or PAI-1 (2.2 nmol/l; 100 ng/ml), followed by the wound-healing assay as described above. Results are given as mean ± SD (*n* = 3). *P < 0.05, NS = not significant, compared with the untreated control. **(D)** BV-2 microglial cells were seeded at the density of 5.0 × 10^3^ cells/well in 96-well plate. Cells were treated with a mouse PAI-1 protein (100 ng/ml) and incubated at 37°C for 12–72 hours to evaluate cell proliferation. Proliferation curves are based on the 2,5-diphenyltetrazolium bromide (MTT) assay. Results represent the mean ± SD (n = 3). Proliferation of PAI-1-treated cells (open circle) was compared with the untreated control (filled square). **(E)** The Boyden chamber assay was performed to evaluate primary microglial cell migration. Primary microglia cultures (5 × 10^4^ cells/upper well) placed in the Boyden chambers were exposed to mouse PAI-1 protein (100 ng/ml), and then incubated at 37°C for 24 hours. Microglial cells that migrated through a membrane were stained and counted. Results are mean ± SD (*n* = 3). *P < 0.05; compared with the untreated control (lower panel). Representative images are also shown (upper panel). **(F)** C6 glioma cells were seeded at the density of 8.0 × 10^4^ cells/well in 96-well plate. Cells were treated with BSA (100 ng/ml) or PAI-1 (100 ng/ml), followed by the wound-healing assay as described above. Results are the mean ± SD (*n* = 3). *P < 0.05; compared with the untreated control.

Having shown that both microglia and astrocytes secrete PAI-1 upon inflammatory stimulation, we next sought to determine how glia-derived PAI-1 influences proinflammatory phenotypes of microglia. We focused on microglial migration, nitric oxide (NO) production, and neurotoxicity, because it has been suggested that activated microglia are recruited to inflammatory sites and produce NO and other proinflammatory mediators, amplifying neuroinflammation and exerting neurotoxic effects. Effects of PAI-1 on microglial cell migration were first investigated using an *in vitro* wound-healing assay (Figure 2A) and Boyden chamber assay (Figure 2B, E). The mean plasma concentration of PAI-1 under physiological conditions is about 6 to 80 ng/ml, but it can be increased in a number of pathological conditions [68]. In the migration assay, we used 1 to 1000 ng/ml of recombinant mouse PAI-1 protein, which is equivalent to 0.022 to 22.0 nmol/l.

We found that PAI-1 promoted migration of BV-2 microglial cells in a dose-dependent manner. Significant effects on microglial migration were seen after treatment with 10 ng/ml (0.22 nmol/l) or higher concentrations of PAI-1 protein. Effects of BSA at the same molar concentration (0.022 to 22.0 nmol/l) were compared as a control (Figure 2C). Sensitivity of microglia to PAI-1 was similar to that of rat and human smooth-muscle cells, MEF-1 fibroblasts, and HT1080 fibrosarcoma cells [35]. PAI-1 did not affect microglial proliferation (Figure 2D), indicating that the PAI-1 promotion of wound recovery was not related to microglial cell proliferation. PAI-1 also increased migration of primary microglia cultures (Figure 2E). These results, taken collectively, indicate that PAI-1 promotes the migration of microglia in culture. PAI-1 also increased C6 rat glioma cell migration by about 1.25-fold over control (Figure 2F), suggesting that PAI-1 may exert similar effects on the dynamics of microglia and astrocytes. However, the effects of PAI-1 on astrocytes were not further investigated in this study.

**Figure 3:**
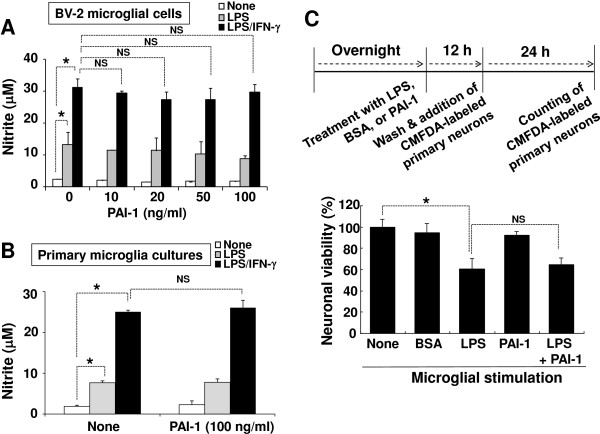
**No significant effects of plasminogen activator inhibitor type 1 (PAI-1) on microglial nitric oxide (NO) production or neurotoxicity after lipopolysaccharide (LPS) or interferon (IFN)-γ stimulation. (A)** BV-2 microglial cells and **(B)** primary microglia cultures were treated with the indicated concentration of mouse PAI-1 protein, LPS (100 ng/ml), and IFN-γ (50 U/ml) for 24 hours. NO production was measured by the Griess reaction. Results are given as mean ± SD (*n* = 3). *P < 0.01, NS = not significant. (**C)** Primary microglia cultures (4.0 × 10^4^ cells/well) were treated for 12 hours with PAI-1 (100 ng/ml), BSA (100 ng/ml), or LPS (100 ng/ml) as indicated. Afterwards, primary microglial cells were cocultured with (upper panel) 5-chloromethyl-fluoresceindiacetate (CMFDA)-labeled primary neuron cultures for 24 hours (co-culture scheme). (Lower panel) CMFDA-positive neurons in the five randomly chosen microscopic fields per well were counted under an inverted microscope. Results are given as mean ± SD (*n* = 3). *P < 0.05, NS = not significant.

Next, we determined whether PAI-1 could directly affect microglial activation. Because activated microglia release NO and other neurotoxic mediators, microglial NO production and neurotoxicity was measured to assess microglial activation. The recombinant mouse PAI-1 protein (10 to 100 ng/ml) did not affect LPS-induced NO production (Figure 3A,B) or cell viability (data not shown) in BV-2 microglial cells or primary microglia cultures. PAI-1 did not influence microglial neurotoxicity in microglia–neuron cocultures (Figure 3C). LPS-stimulated microglia were neurotoxic in the co-culture, and this was not affected by PAI-1. These results indicate that PAI-1 does not affect microglial activation following LPS stimulation.

### Plasminogen activator inhibitor type 1 (PAI-1) promotes microglial migration through the low-density lipoprotein receptor-related protein 1/Janus kinase/signal transducer and activator of transcription-1 pathway

**Figure 4:**
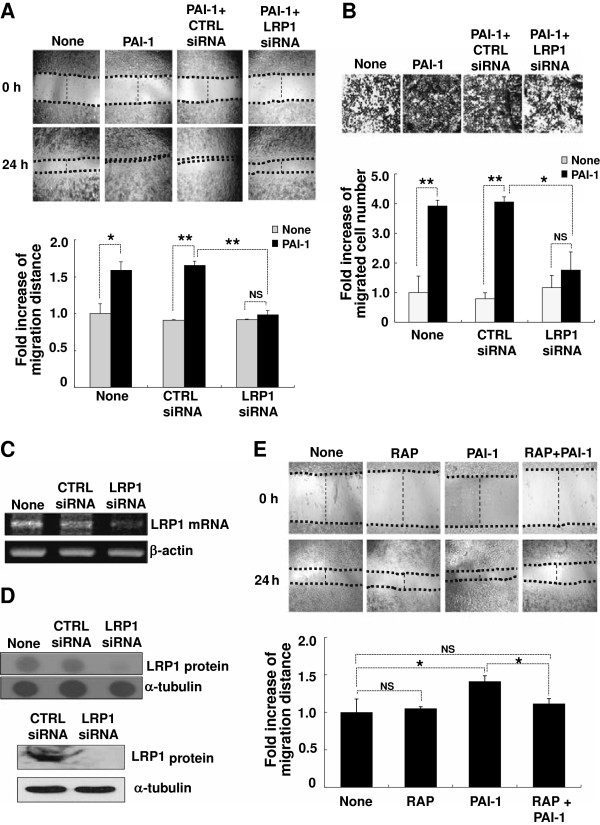
**Plasminogen activator inhibitor type 1 (PAI-1) promoted microglial migration through low-density lipoprotein receptor-related protein (LRP)1. (A, B)** BV-2 microglial cells were transiently transfected with control small interfering (si)RNA or LRP1-specific siRNA. After 48 hours, a scratch wound was made. Cells were treated with or without mouse PAI-1 protein (100 ng/ml), followed by **(A)** the wound-healing assay and **(B)** the Boyden chamber assay, as described in Figure 2. Results are given as mean ± SD (*n* = 3). *P < 0.05, **P < 0.01, NS = not significant (lower panel). (Upper panels) Representative images of each assay. **(C, D)** Knockdown of LRP1 gene expression by siRNA was confirmed by using **(C)** reverse transcriptase PCR, **(D)** dot blotting (upper panel), and western blotting (lower panel). β-actin and the α-tubulin were used as internal controls. **(E)** BV-2 microglial cells were treated with mouse PAI-1 protein (100 ng/ml) and RAP protein (5 μg/ml) as indicated. The fold increase in migration distance was measured using the wound-healing assay. Results are given as mean ± SD (*n* = 3). *P < 0.05, NS = not significant, compared with the untreated control (lower panel). (Upper panel) Representative images also shown.

LRP1 has been previously implicated in the biological functions of PAI-1 [69,70]. LRP1 is a cell surface protein that has been shown to bind to a variety of ligands including apolipoprotein E, lipoprotein lipase, uPA, tPA, and PAI-1 [69]. To determine the role of LRP1 in the PAI-1-mediated microglial cell migration, we used LRP1 siRNA and RAP protein to inhibit LPR1 pathway. RAP has been shown to bind LRP1 and block its interactions with all known ligands including PAI-1. LRP1 gene silencing using siRNA abolished the PAI-1-promoted BV-2 microglial cell migration as determined by the wound-healing assay (Figure 4A) and the Boyden chamber assay (Figure 4B). Knockdown of LRP1 expression was shown by RT-PCR (Figure 4C), dot blotting analysis, and western blotting analysis using an LRP1-specific antibody (Figure 4D). The addition of RAP protein alone did not affect wound closure, but it completely blocked the migration-enhancing effect of PAI-1 in the wound-healing assay (Figure 4E). RAP was also able to block the effect of PAI-1 (1–100 ng/ml) in the Boyden chamber assay (data not shown). These results show that PAI-1 stimulates microglial migration via LRP1.

**Figure 5:**
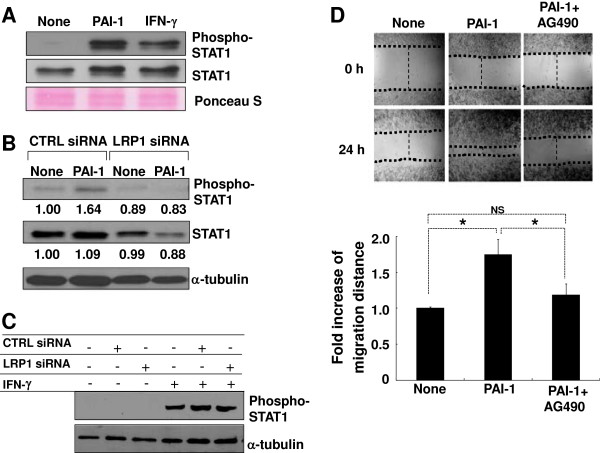
**Janus kinase (JAK)/signal transducer and activator of transcription (STAT)-1 was involved in the plasminogen activator inhibitor type 1 (PAI-1)-enhanced microglial motility. (A)** BV-2 microglial cells were treated with mouse PAI-1 protein (100 ng/ml) or interferon (IFN)-γ (50 U/ml), and cell lysates were collected at 30 minutes after the treatment. The levels of phosphorylated STAT1 (pSTAT1 at Tyr701) or total STAT1 protein were then evaluated by western blotting analysis. Ponceau S staining was performed to confirm the equal loading of the samples. **(B)** BV-2 microglial cells were transfected with control small interfering (si)RNA or low-density lipoprotein receptor-related protein (LRP)1 siRNA. The cells were harvested 48 hours after transfection and used for the experiments. Cells were treated with mouse PAI-1 protein (100 ng/ml) for 30 minutes. Phosphorylated STAT1 or total STAT1 was measured by western blotting analysis. α-tubulin detection was also performed to confirm the equal loading of the samples. Values indicate the results of densitometric quantification normalized to α-tubulin. **(C)** BV-2 microglial cells were transfected with control siRNA or LRP1 siRNA. The cells were harvested at 48 hours after transfection and then treated with mouse IFN-γ (50 U/ml) for 30 minutes. Phosphorylated STAT1 was detected by western blotting analysis. α-tubulin detection was also performed to confirm the equal loading of the samples. **(D)** BV-2 microglial cells were pretreated with AG490 (JAK-specific inhibitor; 20 μmol/l) for 30 minutes before the treatment with mouse PAI-1 protein (100 ng/ml), and then cell migration was evaluated by the wound-healing assay. Results are mean ± SD (*n* = 3). *P < 0.05, NS = not significant, compared with the untreated control (lower panel). Representative images are shown (upper panel; original magnification × 150).

We next addressed intracellular signaling pathways associated with the PAI-1 activity. The JAK/STAT pathway has been previously implicated in cell migration [71,72], and a previous study [35] has shown that PAI-1 stimulates STAT1 activation in rat smooth-muscle cells. Thus, we evaluated the role of JAK/STAT1 pathway in the PAI-1-promoted microglial cell migration after LRP1 binding. PAI-1 alone induced STAT1 phosphorylation as determined by western blotting in BV-2 microglial cells (Figure 5A). IFN-γ was used for comparison purposes [73]. LRP1 gene silencing diminished PAI-1-induced STAT1 phosphorylation (Figure 5B). LRP siRNA did not reduce IFN-γ-induced STAT1 phosphorylation, indicating that LRP siRNA did not cause cell toxicity (Figure 5C). Thus, LRP1 knockdown inhibited PAI-1-induced STAT1 expression and activation. These results indicate that PAI-1 promotes microglial migration through the JAK/STAT1 pathway, and that LRP1 may reside in the upstream of the JAK/STAT1 signaling pathway in microglia. Indeed, the addition of AG490, a pharmacological inhibitor of JAK kinase, significantly attenuated the PAI-1-induced BV-2 microglial cell migration in the wound-healing assay (Figure 5D). These data indicate that PAI-1 enhances microglial cell migration via LRP1 and the JAK/STAT1 pathway.

### Plasminogen activator inhibitor type 1 (PAI-1) is an inducer of microglial migration *in vivo*

**Figure 6:**
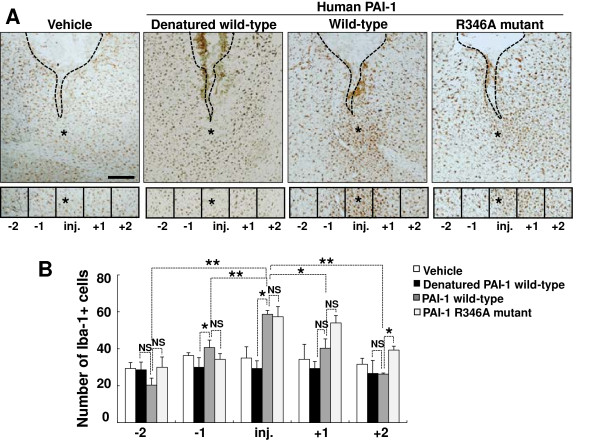
**Plasminogen activator inhibitor type 1 (PAI-1) promoted microglial migration*in vivo*. (A, B)** Microglial cells around the protein injection sites were stained with anti-Iba-1 antibody at 48 hours after intrastriatal injection of vehicle (dialyzed elution buffer that was used for PAI-1 protein purification), denatured human PAI-1 wild-type, PAI-1 wild-type, or PAI-1 R346A mutant protein (1 μl; 1.5 μg/μl). Boxes indicate the 300 × 300 μm squares placed for cell counting. Immunohistochemistry results showed that Iba-1-positive cells were recruited into the injection site after injection of wild-type or R346 mutant PAI-1 protein. **(B)** Results are given as mean ± SD from three animals and six independent sections per animal. *P < 0.05, **P < 0.01, NS = not significant. **(A)** Representative images (scale bar, 300 μm). Asterisks indicate the injection sites (inj.). Guide cannula was stereotaxically located in the intrastriatal region (dotted line).

**Figure 7:**
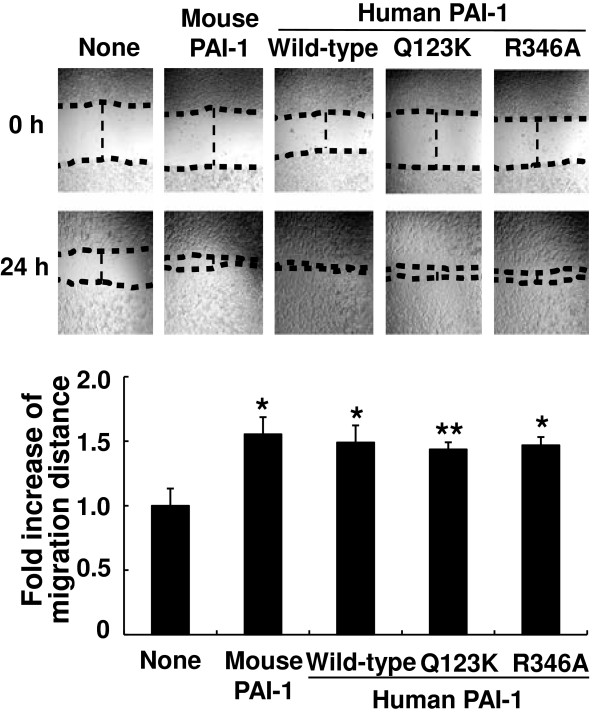
**Plasminogen activator inhibitor type 1 (PAI-1)-induced microglial migration was independent of fibrinolysis or vitronectin binding.** BV-2 microglial cells were treated with 100 ng/ml of mouse PAI-1, human wild-type PAI-1, or two variants (Q123K, R346A) of PAI-1 proteins, followed by a wound-healing assay as described in Figure 2. The wound recovery areas were visualized under an inverted microscope (upper panel), and the fold increase in migration distance was measured. Results are given as mean ± SD (*n* = 3). *P < 0.05, **P < 0.01; compared with the untreated control (lower panel).

**Figure 8:**
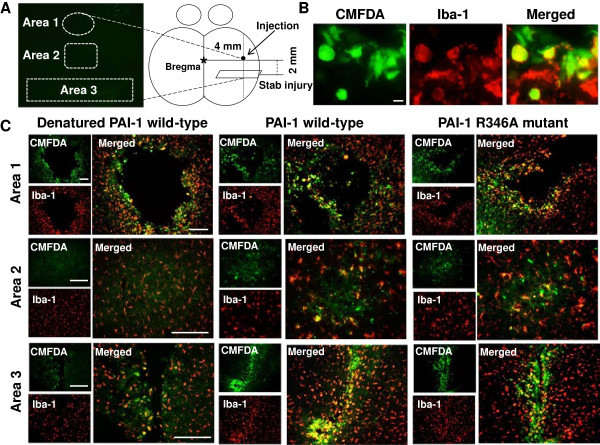
**Plasminogen activator inhibitor type 1 (PAI-1) enhanced 5-chloromethyl-fluoresceindiacetate (CMFDA)-labeled microglial migration*in vivo.*** The effect of PAI-1 on microglial migration was examined using a stab-injury/cell-injection model. Primary microglia were treated with 1 μg/ml of PAI-1 protein (denatured wild-type protein (control), wild-type protein, R346A mutant protein) for 12 hours. Microglial cells were then labeled with CMFDA and injected into the mouse brain. **(A)** After 72 hours, three areas (cell-injection site (area 1), an intermediate location between the injection site and the stab-injury site (area 2), and the stab-injury site (area 3) were chosen for the analysis of CMFDA-labeled microglial cell migration (left panel). **(A)** A schematic diagram of the stab-injury/cell-injection model is also shown (right panel). The stab injury (3 mm long, 2 mm deep) was created 2 mm posterior to the bregma and 4 mm right lateral to the midline. **(B)** Representative images of CMFDA (green) and Iba-1 (red) staining. Scale bar, 20 μm. **(C)** Iba-1 microglial staining in the three areas of brain. Scale bar, 200 μm.

To determine whether PAI-1 promotes microglial motility *in vivo*, microglial accumulation was investigated after intrastriatal injection of human PAI-1 protein. Vehicle, denatured wild-type human PAI-1, wild-type human PAI-1, or the R346A human PAI-1 protein mutant were stereotaxically injected into the striatum of the mouse brain. Accumulation of microglia was immunohistochemically evaluated by counting Iba-1-positive cells around the injected area. At 48 hours after intrastriatal injection of wild-type human PAI-1 protein, there were large numbers of Iba-1-positive microglia accumulated around the PAI-1 injection site (Figure 6A,B). The R346A mutant protein, which is not capable of inhibiting PA, similarly induced microglial accumulation around the injection site. Denatured PAI-1 protein had no effect. Because the injection alone may cause tissue injuries, a basal level of microglial accumulation was seen after vehicle injection. Because PAI-1 did not induce microglial activation *in vitro* (Figure 3A,B), we suggest that the microglial accumulation seen in this experiment probably results from microglial recruitment rather than activation. The microglial migration-promoting activity of the R346A mutant protein was also seen in an *in vitro* migration assay, indicating that the PAI-1 effects are independent of the fibrinolysis system (Figure 7). Additionally, the Q123K mutant of human PAI-1 retained the migration-promoting activity *in vitro*, thereby suggesting that binding of PAI-1 to vitronectin may not be required for the activity. Recombinant human PAI-1 protein has been shown previously to be effective in mice [74]. Indeed, human and mouse PAI-1 protein exerted similar effects on the stimulation of microglial migration (Figure 7). To further exclude the possibility that microglial accumulation around the injection site is not due to cell activation or proliferation, another *in vivo* migration assay was performed using a stab-injury/cell-injection model, which has been previously used to determine glial cell migration *in vivo*[57,58]. In this method, fluorescently labeled microglial cells were injected into the cortex, and their migration toward the stab-injury site monitored. For this, primary microglial cells were treated with 1 μg/ml of PAI-1 protein (denatured wild-type protein as a control, wild-type protein, R346A mutant protein) for 12 hours, and the cells labeled with CMFDA. The CMFDA-labeled microglial cells were injected into the mouse brain, and then the stab injury was created. After 72 hours, three different areas (the cell-injection site, a location intermediate between the injection site and the stab-injury site, and the stab-injury site) were visible (Figure 8). Iba-1 immunostaining was also performed to identify microglial cells. Iba-1/CMFDA double-labeled cells were accumulated around the stab-injury site in the mouse brains after injection with PAI-1 wild-type or R346A mutant protein-treated microglia. Denatured PAI-1 protein had no effect. The results support the notion that PAI-1 promotes microglial migration *in vivo*.

### Plasminogen activator inhibitor type 1 (PAI-1) derived from astrocytes regulated microglial migration

**Figure 9:**
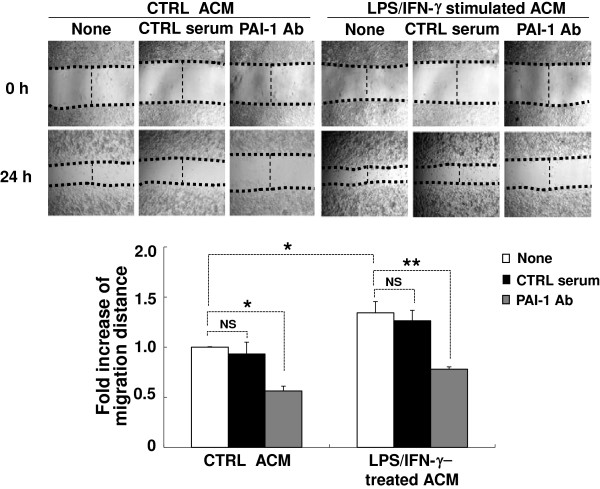
**Astrocyte-derived plasminogen activator inhibitor type 1 (PAI-1) promoted the migration of microglia.** Primary astrocytes were left untreated or treated with lipopolysaccharide (LPS; 100 ng/ml) and interferon (IFN)-γ (50 U/ml) for 12 hours. Cells were then washed twice with PBS, and cultured in fresh DMEM for an additional 24 hours. The astrocyte-conditioned medium (ACM) was then collected. BV-2 microglial cells were treated for 24 hours with ACM in the presence or absence of PAI-1 neutralizing antibody (PAI-1 Ab; 2 μg/ml), or normal rabbit serum (2 μg/ml) as control. Microglial migration was assessed by wound-healing assay as described in Figure 2. At 0 and 24 hours, phase-contrast images of the wounds at three different locations were taken (upper panel, original magnification, × 150), and then fold increase in migration distance from three independent experiments was measured (lower panel). Results are given as mean ± SD (*n* = 3). *P < 0.05, **P < 0.01, NS = not significant.

In a series of experiments, we presented evidence that addition of exogenous PAI-1 protein promotes microglial migration both *in vitro* and *in vivo*. We next aimed to determine the role of endogenous PAI-1 protein in the regulation of microglial migration. Although microglia may contribute to PAI-1 secretion, astrocytes are thought to be the major cellular source of PAI-1 in the CNS *in vivo*[23,43,75,76], because astrocytes outnumber microglia in the brain. Astroglial PAI-1 release was also detected in the current study (Figure 1B). Thus, we assessed the role of astrocyte-derived PAI-1 in the regulation of microglial migration using ACM and neutralizing antibodies against PAI-1. ACM was prepared from primary astrocyte cultures stimulated with a combination of LPS and IFN-γ. ACM promoted the migration of BV-2 microglial cells as determined by the wound-healing assay (Figure 9). To neutralize the PAI-1 activity in the ACM, a polyclonal anti-PAI-1 antibody was applied to BV-2 microglial cells together with ACM. Normal rabbit serum was used as a control. Abolishment of PAI-1 activity using anti- PAI-1 antibody significantly inhibited the effect of LPS/IFN-γ-stimulated ACM on microglial migration (Figure 9). PAI-1 neutralization also attenuated the effect of unstimulated ACM, indicating the presence of a low concentration of PAI-1 in the control ACM. These results further support that PAI-1 plays an important role in neuroinflammation by promoting microglial migration.

### Plasminogen activator inhibitor type 1 inhibited microglial phagocytosis of zymosan particles

**Figure 10:**
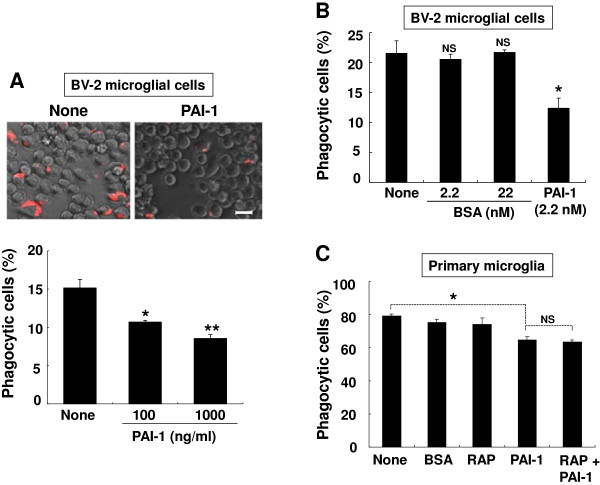
**Plasminogen activator inhibitor type 1 (PAI-1) inhibited microglial phagocytosis of zymosan particles. (A)** BV-2 microglial cells were treated with PAI-1 (100 or 1000 ng/ml) for 1 hour and then incubated with zymosan particles conjugated with Alexa Fluor 594 (red) for 3 hours. Cells were washed five times with ice-cold PBS to remove bound particles. **(A)** Images at five different locations were taken and then the percentage of phagocytic cells was calculated based on the number of microglial cells that phagocytosed the zymosan particles (lower panel). (Upper panel) Representative images are shown. Results are given as mean ± SD from three independent experiments. *P < 0.05, **P < 0.01, different from untreated control. Scale bar = 20 μm. **(B)** BV-2 microglial cells were treated with BSA (2.2 or 22 nmol/l) or PAI-1 (2.2 nmol/l; 100 ng/ml) for 1 hour and then incubated with zymosan particles for 3 hours, followed by the phagocytosis assay as described above. Results are given as mean ± SD from three independent experiments. *P < 0.05, NS = not significant, compared with the untreated control. Scale bar = 20 μm. **(C)** Primary microglia were treated with BSA (100 ng/ml), PAI-1 (100 ng/ml), or RAP protein (5 μg/ml) for 1 hour and then incubated with zymosan particles for 90 minutes. Microglial phagocytosis was assessed as described above. Results are given as mean ± SD (*n* = 3). *P < 0.05, NS = not significant.

**Figure 11:**
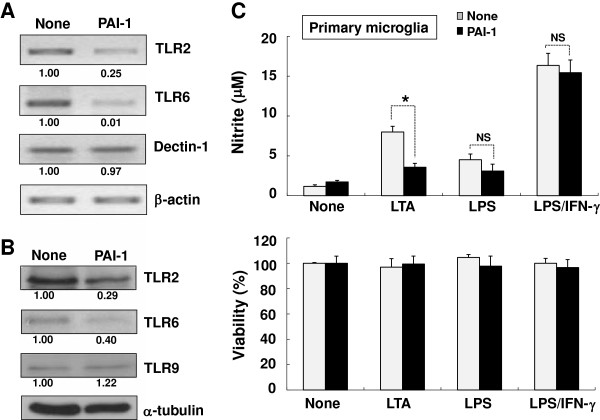
**Plasminogen activator inhibitor type 1 (PAI-1) downregulated Toll-like receptor (TLR)2/6 expression and its signaling. (A)** BV-2 microglial cells were treated with PAI-1 (100 ng/ml) for 5 hours. TLR2/TLR6 and dectin-1 gene expression was detected by reverse transcriptase PCR. β-actin was used as an internal control. **(B)** Alternatively, BV-2 microglial cells were treated with PAI-1 (100 ng/ml) for 24 hours. The levels of TLR2, TLR6, and TLR9 protein were then evaluated by western blotting analysis. α-tubulin was used as an internal control. Values indicate the results of densitometric analysis normalized to either β-actin or α-tubulin. **(C)** Primary microglia cultures were treated with mouse PAI-1 protein (100 ng/ml), lipopolysaccharide (LPS; 100 ng/ml), interferon (IFN)-γ; 50 U/ml), and lipoteichoic acid (LTA; 1 μg/ml) as indicated for 24 hours. (Upper panel) NO production was measured by a Griess reaction. (Lower panel) Cell viability was measured by 2,5-diphenyltetrazolium bromide (MTT) reduction assays, and the results expressed as the percentage of surviving cells over the control cells . Results are given as mean ± SD (*n* = 3). *P < 0.01, NS = not significant.

**Figure 12:**
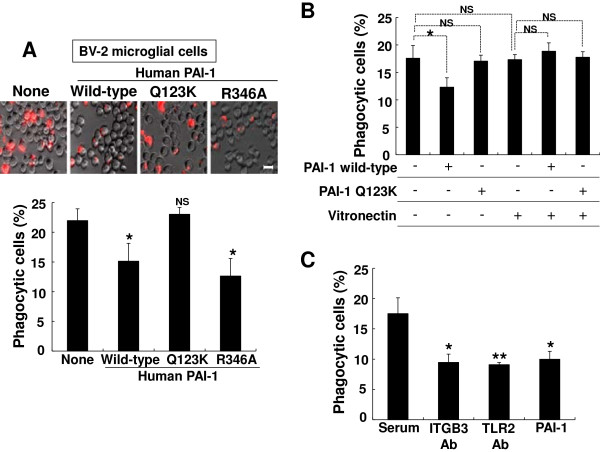
**Plasminogen activator inhibitor type 1 (PAI-1) inhibited microglial phagocytosis in a vitronectin-dependent manner. (A)** BV-2 microglial cells were treated with 100 ng/ml of human wild-type PAI-1, the Q123K variant, or the R346A variant for 1 hour, and then incubated for 3 hours with zymosan particles. **(A)** Microglial phagocytosis of fluorescent zymosan particles was assessed (lower panel) as described above. (Upper panel) Representative images are shown. Results are given as mean ± SD (*n* = 3). *P < 0.05, NS = not significantly different from untreated control. Scale bar = 20 μm. **(B)** BV-2 microglial cells were treated with human wild-type PAI-1 or the Q123K mutant (100 ng/ml) in the presence or absence of vitronectin (1 μg/ml) for 1 hour, and then incubated with zymosan particles for 3 hours, followed by phagocytosis assay. Results are given as mean ± SD (*n* = 3). *P < 0.05, NS = not significant. **(C)** BV-2 microglial cells were incubated with zymosan particles in the presence or absence of Toll-like receptor (TLR)2 antibody (TLR2 Ab; 2 μg/ml), integrin (ITG)B3 antibody (ITGB3 Ab; 2 μg/ml), normal rabbit serum (2 μg/ml; negative control), or PAI-1 (100 ng/m; positive control). Microglial phagocytosis assay was performed. Results are given as mean ± SD (*n* = 3). *P < 0.05, **P < 0.01, compared with normal serum.

The effect of PAI-1 protein on the phagocytic activity of microglia was next investigated using zymosan particles as a prey. Zymosan particles are components of yeast cell wall, and served as a model for the phagocytosis of invading microbes [77]. The recombinant mouse PAI-1 protein inhibited the engulfment of zymosan particles in both BV-2 microglial cells and primary microglia cultures (Figure 100A, C). PAI-1 inhibited the microglial phagocytic activity in a dose-dependent manner, as 1000 ng/ml of PAI-1 treatment produced greater inhibition than 100 ng/ml (Figure 10A). BSA (2.2 or 22.0 mol/l) did not inhibit the phagocytic activity of microglia (Figure 10B). To identify the role of LRP1 in the PAI-1 inhibition of microglial phagocytosis, primary microglial cultures were treated with PAI-1 in the presence of RAP peptide (Figure 10C). The addition of RAP did not affect the PAI-1 inhibition of microglial phagocytic activity, indicating that LRP1 is not involved in the PAI-1 reduction of microglial phagocytosis. TLR2, TLR6 and glucan receptor dectin-1 have been previously implicated in the recognition and phagocytosis of zymosan particles in either a cooperative or independent manner [78,79]. The mRNA (Figure 11A) and protein (Figure 11B) levels of TLR2 and TLR6 were markedly decreased after 6 hours of PAI-1 treatment, but there was no significant difference in dectin-1 mRNA or TLR9 protein levels (Figure 11A, B). Consistent with TLR2 mRNA/protein reduction, PAI-1 inhibited TLR2-mediated microglial activation as determined by NO production after stimulation with the TLR2 agonist LTA in primary microglia cultures (Figure 11C). To further define the inhibitory mechanism of PAI-1 in microglial phagocytosis, we used wild-type human PAI-1 protein, and the R346A and Q123K mutants of this protein. The wild-type protein and the R346A mutant (unable to inhibit PA) inhibited the engulfment of zymosan particles, whereas the Q123K mutant (with impaired vitronectin binding activity) did not have an inhibitory effect (Figure 12A). The addition of recombinant vitronectin protein to PAI-1-treated microglial cells rescued the phagocytic activity (Figure 12B). We speculate that PAI-1 may inhibit the engulfment of zymosan particles by interfering with vitronectin/ITGB3 interaction. Vitronectin is a multifunctional molecule that binds to PAI-1, ITGB3, and bacteria [80]. To verify our hypothesis, the anti-TLR2 or anti-ITGB3 antibodies were applied to BV-2 microglial cells together with zymosan particles. Neutralization of either TLR2 or ITGB3 significantly inhibited microglial phagocytosis. The percentage inhibition by anti-TLR2 or anti-ITGB3 antibody was similar to that of recombinant PAI-1 (Figure 12C). These results suggest that PAI-1 may inhibit microglial phagocytic activity via TLR2 and ITGB3.

## Discussion

Stimulated glial cells release various proinflammatory proteins such as cytokines, chemokines, and neurotoxic factors under pathological conditions [1,2]. These soluble proteins may play important roles in the progression of inflammatory diseases. Secretomic analysis of glia has been previously used to determine the secreted protein profiles during inflammatory responses [3,4,6,51,65,66,81]. In this study, we found that PAI-1 is one of the major proteins released by mixed glial cultures after inflammatory stimulation, and we provide evidence that PAI-1 is able to regulate microglial activation, migration, and phagocytosis under inflammatory condition*.*

PAI-1 is the primary inhibitor of uPA and tPA, which are involved in fibrinolysis [21,82]. PAI-1 also exerts numerous effects that are not dependent on PA inhibition [27,28,83]. PAI-1 levels are increased in brain diseases such as glioma, hypoxia, ischemic stroke, MS, and AD [38-41]. Astrocytes, but not microglia, are thought to be the major cellular source of PAI-1 in the CNS *in vivo*[23,43,75,76]. Our data suggest that microglia can also be a source of PAI-1 in the CNS. A recent study indicates that PAI-1 is also expressed in olfactory ensheathing glia [44]. In the current study, PAI-1 mRNA expression was detected in primary astrocytes, primary microglia cultures, and cell lines of microglia or astrocyte origin (Figure 1C,D). PAI-1 protein secretion was increased in the LPS/IFN-γ-stimulated primary microglia and astrocyte cultures (Figure 1B). Thus, PAI-1 secreted by microglia or astrocytes may regulate microglial motility and phagocytic activity in an autocrine or paracrine manner under inflammatory conditions. Because microglial activation and ensuing neuroinflammation are key components of neurodegenerative diseases such as AD, PD, and MS, PAI-1 is likely to play an important role in regulating the inflammatory activation of microglia. Microglia-mediated neuroinflammation is characterized by a series of events, with a crucial step being the migration of microglia to the site of brain injury or inflammation [2,84], of which PAI-1 seems to be a central regulator. We found that PAI-1 modulates microglial activation after stimulation with TLR2 (Figure 11C), but not TLR4 (Figure 3A,B). TLR2 has been previously shown to exacerbate ischemic brain damage [85]. PAI-1 may play a regulatory role under pathological condition by suppressing TLR2 signaling. Indeed, PAI-1 has been shown to prevent apoptosis and even to protect against brain injury [42].

PAI-1 has been previously implicated in cell migration [86], and regulates cell migration through multiple mechanisms. PAI-1 has been shown to either enhance or suppress cell migration by interacting with various partner proteins such as uPA, tPA, LRP1, and vitronectin. PAI-1 suppresses cell migration by binding to vitronectin or uPA/uPAR. PAI-1 inhibited the motility of vascular smooth-muscle cells, human amnion WISH cells, and carcinoma cells via interaction with vitronectin [28-30,87], and vitronectin blocked the LRP1/PAI-1 pathway [33]. The PAI-1/uPA/uPAR complex inhibited uPA-induced cell migration [34], whereas this complex mediated vitronectin-induced cell migration [88]. PAI-1 has been implicated in cancer invasion [89,90] and angiogenesis [90]. PAI-1 stimulated the migration of monocytes and macrophages by interacting with LRP or tPA. By binding to LRP1, PAI-1 also enhanced the migration of rat and human smooth-muscle cells, mouse embryonic fibroblast-1, and fibrosarcoma cells (HT1080) [35]. PAI-1 also promoted the migration of lymphocytes and neutrophils into inflammatory sites [35,91,92]. Deficiency of PAI-1 abolished the migration of exudate macrophages [93]. The LRP/tPA/PAI-1 complex coordinated Mac-1-dependent macrophage migration [37]. In the previous studies, the regulatory effects of PAI-1 on cell migration have been shown in various cell types such as monocytes and endothelial cells. However, it is not clear whether PAI-1 has positive or negative effects on glial cell migration in the CNS. The composition of the extracellular matrix (ECM) in the CNS is different from that of other tissue types. Laminin, fibronectin, and collagen are the major components of the ECM in most tissues, but are largely undetectable in the CNS [94-96]. Because the effect of PAI-1 heavily depends on ECM components such as vitronectin, PAI-1 may not necessarily play the same role in the CNS as in other peripheral tissues. In this study, we found that PAI-1 exerts positive effects on cell migration in the CNS. PAI-1 stimulated microglial migration through the LRP-1/JAK/STAT axis (Figure 4; Figure 5), which is consistent with previous reports in which STAT1 activation was found to be involved in PAI-1 induced cell migration in rat and human smooth-muscle cells and fibroblasts [35]. We used two different PAI-1 mutants to further characterize the cell migration-promoting activity of PAI-1. Vitronectin, in addition to PA, has been identified as a PAI-1-binding protein. The Q123K and R346A mutants, which, respectively, are unable to bind to vitronectin and unable to inhibit PA, retained the microglial migration-promoting activity (Figure 6, Figure 7, Figure 8). These results suggest that the microglia migration-regulating activity of PAI-1 we observed in the current study may not depend on either vitronectin binding or PA inhibition.

Recent reports indicated a novel role of PAI-1 in the regulation of phagocytosis of apoptotic or viable cells [27,97]. Our results show that PAI-1 inhibits microglial phagocytosis of zymosan particles (Figure 10 A,C). Human PAI-1 proteins (both wild-type and the R346A mutant) inhibited microglial phagocytic activity, whereas the Q123K mutant (unable to bind to vitronectin) did not. These results prompted us to speculate that PAI-1 inhibits microglial phagocytosis by binding to vitronectin, which is a functional partner of PAI-1. The PAI-1/vitronectin complex interacts with the Arg-Gly-Asp motif of ITGB3, inhibits fibrinolysis, and modulates the pro-migratory effect of PAI-1 [28,30,32,33,87]. Vitronectin and integrin were previously shown to be required for TLR2-mediated activation of monocytes [98], and zymosan phagocytosis was dependent on TLR2 and TLR6 [99-101], while TLR2 deficiency attenuated bacterial clearance [102]. Our results suggest that PAI inhibits microglial phagocytosis by blocking the vitronectin/ITGB3/TLR2 complex. Indeed, neutralization of ITGB3 or TLR2 inhibited microglial phagocytosis (Figure 12C). We also found that PAI-1 inhibited TLR2 and TLR6 expression (Figure 11A,B). Thus, PAI-1-mediated downregulation of TLR2 seems to reduce microglial phagocytic activity.

## Conclusions

In this study, we found that PAI-1 released from microglia and astrocytes promotes microglial migration and inhibits phagocytosis *in vitro*. Some of our *in vitro* findings were supported by animal studies, in which PAI-1 was found to stimulate microglial recruitment into the injury site in mouse brain. PAI-1 promoted microglial migration via the LRP1/JAK/STAT1 axis, and inhibited microglial phagocytosis of zymosan particles. Extensive studies have been conducted for PAI-1 in cardiovascular diseases, obesity, and diabetes [103-107], but little is known about its role in inflammatory diseases of the brain. Our results suggest PAI-1 as a potential therapeutic target to control microglial migration and phagocytosis under pathological conditions in the CNS.

## Abbreviations

ACM, Astrocyte-conditioned medium; AD, Alzheimer’s disease; BSA, Bovine serum albumin; CMFDA, 5-chloromethyl-fluoresceindiacetate; CNS, Central nervous system; DMEM, Dulbecco’s modified Eagle’s medium; ECM, Extracellular matrix; FBS, fetal bovine serum; GFAP, Glial fibrillary acidic protein; Iba-1, Ionized calcium binding adaptor molecule 1; IFN-γ, Interferon-γ; IPTG, Isopropyl β-D-1-thiogalactopyranoside; ITGB3, Integrin β3; JAK, Janus kinase; LC-MS/MS, Liquid chromatography and tandem mass spectrometry; LPS, Lipopolysaccharide; LRP1, Low-density lipoprotein receptor-related protein 1; LTA, Lipoteichoic acid; MS, Multiple sclerosis; Ni-NTA, Nickel-nitrilotriacetic acid; NO, Nitric oxide; PAI-1, Plasminogen activator inhibitor type 1; PBS, Phosphate-buffered saline; PD, Parkinson’s disease; RAP, Low-density lipoprotein receptor-associated protein; si, Small interfering; STAT1, Signal transducer and activator of transcription-1; tPA, Tissue type plasminogen activators; uPA, Urokinase type plasminogen activators.

## Competing interests

The authors declare that they have no competing interests.

## Authors’ contributions

HJ performed the experiments, analyzed the data, and wrote the manuscript. JHK and JHK both participated in immunohistological investigations and analysis of the data. WHL and MSL participated in the study design and data interpretation. KS was the main investigator of this work, and was in charge of the study design, analysis and interpretation of results, and writing. All authors read and approved the final manuscript.
